# Supplementary material for: Requirement of GrgA for Chlamydia infectious progeny production, optimal growth, and efficient plasmid maintenance
Source: mBio. 2023 Dec 19;15(1):e02036-23. doi: 10.1128/mbio.02036-23 (PMC10790707; doi:10.1128/mbio.02036-23)
Supplement: Figure S2 — Optimization of the ATC-inducible expression system for DOPE of GrgA. [file mbio.02036-23-s0002.pdf]

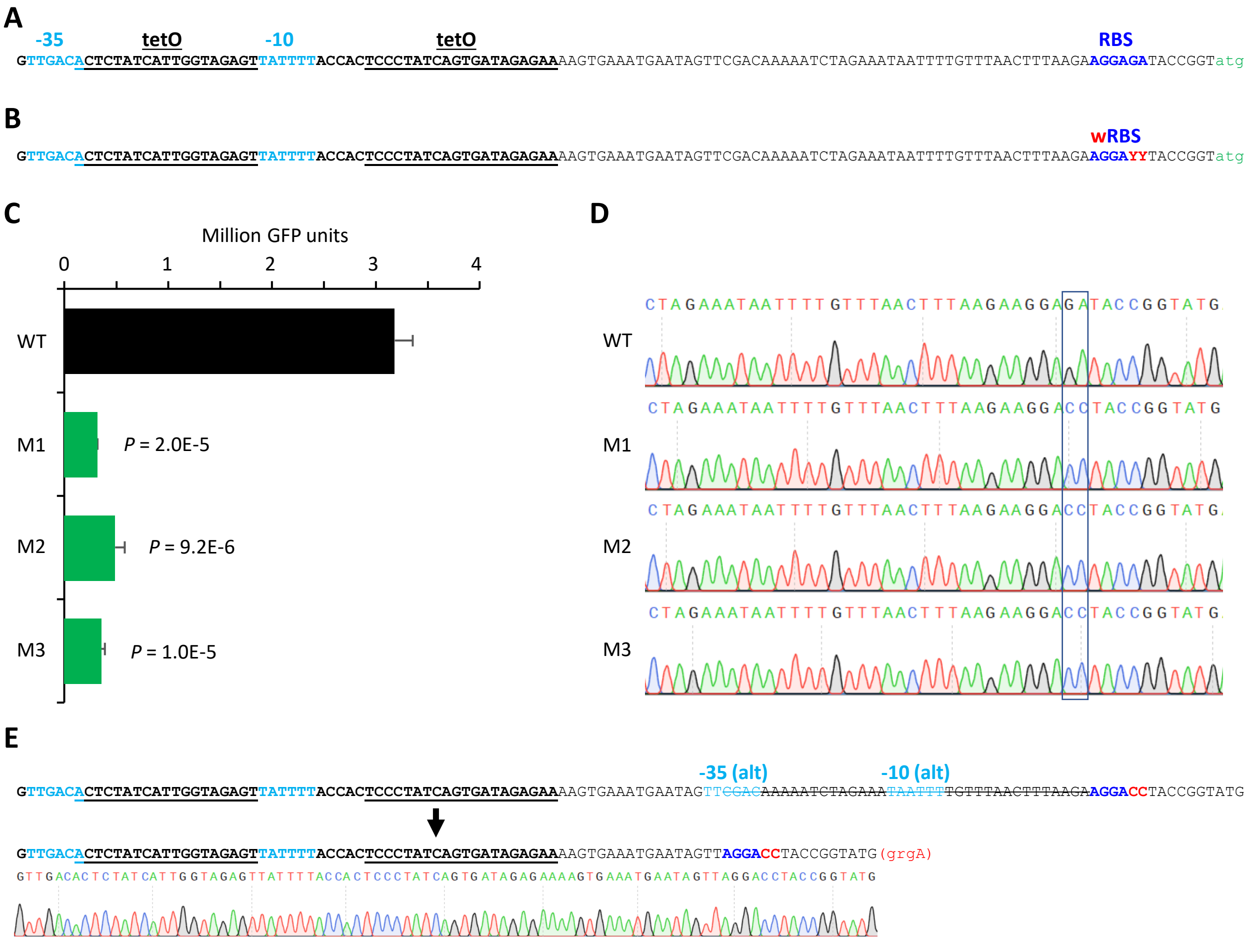

sFigure 2. Optimization of the ATC-inducible expression system for DOPE of GrgA. (A) Nucleotide sequence of the ATC-controlled promoter (Ptet), ribosomal binding site (RBS) and the intervening sequence. (B) Degenerate primer-mediated PCR mutagenesis was used to introduce mutations to the RBS upstream of a green fluorescence protein gene (gfp) in the plasmid pASKGFP/mKate2-L2 (Wickstrum, J., Sammons, L. R., Restivo, K. N. & Hefty, P. S. Conditional gene expression in *Chlamydia trachomatis* using the ATC-inducible system system. PLoS One 8, e76743. https://doi.org:10.1371/journal.pone.0076743). (C) Compared with *E. coli* transformed with parental pASKGFP/mKate2-L2 plasmid, three clones (M1, M2, and M3) of *E. coli* transformed with library plasmid described in B showed reduced GFP expression. Overnight lysogeny broth cultures were diluted 100-fold with the minimal M9 medium containing 2 nM ATC. GFP expression levels were determined using a SpectraMax iD5 plate. (D) Sanger sequencing tracings for plasmids isolated from M1, M2, and M3. Mutated nucleotides and corresponding wildtype nucleotides are boxed. (E) Possible alternative promoter elements between Ptet and RBS were eliminated. The final promoter-RBS fragment was confirmed by Sanger sequencing and used to control GrgA expression from pGrgA-DOPE.
