# Supplementary material for: Requirement of GrgA for Chlamydia infectious progeny production, optimal growth, and efficient plasmid maintenance
Source: mBio. 2023 Dec 19;15(1):e02036-23. doi: 10.1128/mbio.02036-23 (PMC10790707; doi:10.1128/mbio.02036-23)
Supplement: Figure S3 — ATC-induced GrgA expression from pGrgA-DOPE in L2/cg-peig does not affect chlamydial growth. [file mbio.02036-23-s0003.pdf]

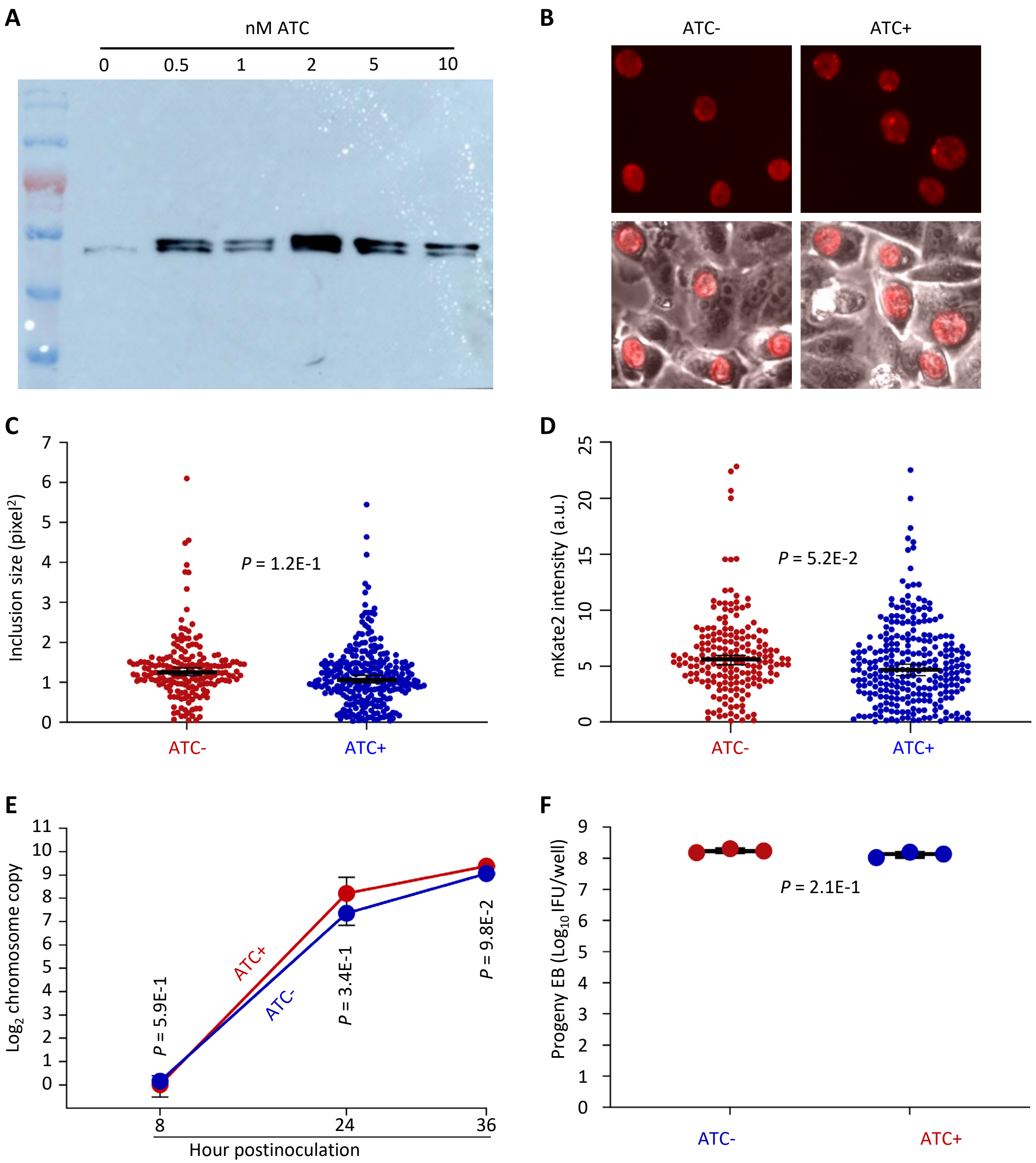

sFigure 3. ATC-induced GrgA expression from pGrgA-DOPE in L2/cg-peig (i.e., C. trachomatis L2 with intact chromosomal grgA) does not affect chlamydial growth. (A) NH-GrgA was detected in cultures containing 0.5 – 10 nM but not 0 nM ATC. Upper and lower bands are plasmid-expressed His-GrgA and chromosome-expressed endogenous GrgA, respectively. HeLa cells infected with L2/cg-peig were cultured with indicated concentrations of ATC in the media. Cultures were terminated at 12 hpi for Western blotting analysis using a polyclonal GrgA antibody that recognizes both the endogenous GrgA and NH-GrgA. (B) Representative images of mKate-expressing inclusions in live cultures of L2/cg-peig containing 0 and 1 nM ATC at 30 hpi. Upper images show mKate signals only (upper), and lower images show mKate-positive inclusions inside host cells photographed under white light. (C) and (D) show quantitative sizes of mKate-expressing inclusions and mKate intensities of the inclusions, respectively. (E) Chlamydial replication kinetics in ATC-containing and ATC-free cultures determined with ctl0631 qPCR. (F) Progeny EBs produced from ATC-containing and ATC-free cultures.
