# Supplementary material for: Requirement of GrgA for Chlamydia infectious progeny production, optimal growth, and efficient plasmid maintenance
Source: mBio. 2023 Dec 19;15(1):e02036-23. doi: 10.1128/mbio.02036-23 (PMC10790707; doi:10.1128/mbio.02036-23)
Supplement: Figure S4 — Confirmation of upregulated euo and hrcA expression in response to GrgA deficiency during midcycle. [file mbio.02036-23-s0004.pdf]

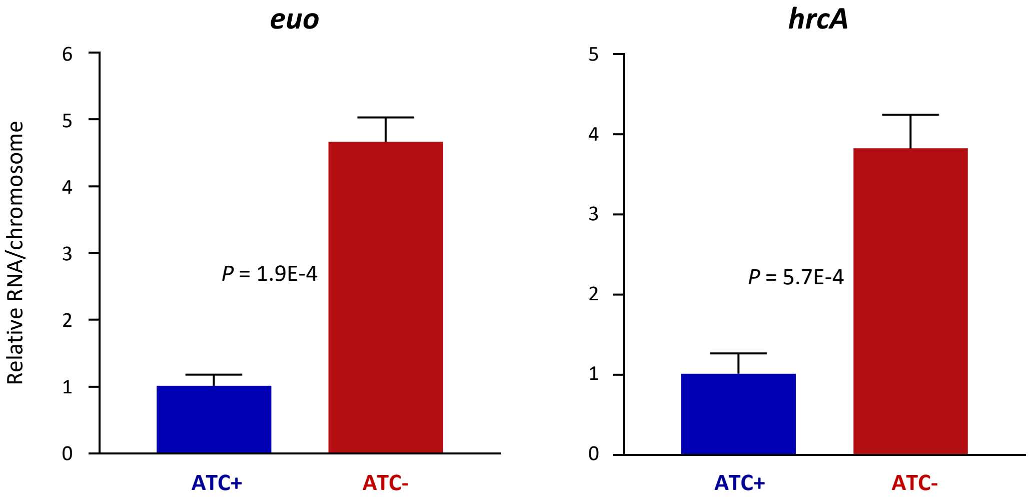

sFigure 4. Confirmation of upregulated euo and hrcA expression in response to GrgA deficiency during midcycle. Presented are qRT-PCR data (averages ± standard deviations) obtained from biological triplicates.
