# Supplementary material for: Requirement of GrgA for Chlamydia infectious progeny production, optimal growth, and efficient plasmid maintenance
Source: mBio. 2023 Dec 19;15(1):e02036-23. doi: 10.1128/mbio.02036-23 (PMC10790707; doi:10.1128/mbio.02036-23)
Supplement: Figure S1 — Map of the shuttle plasmid pGrgA-DOPE. [file mbio.02036-23-s0001.pdf]

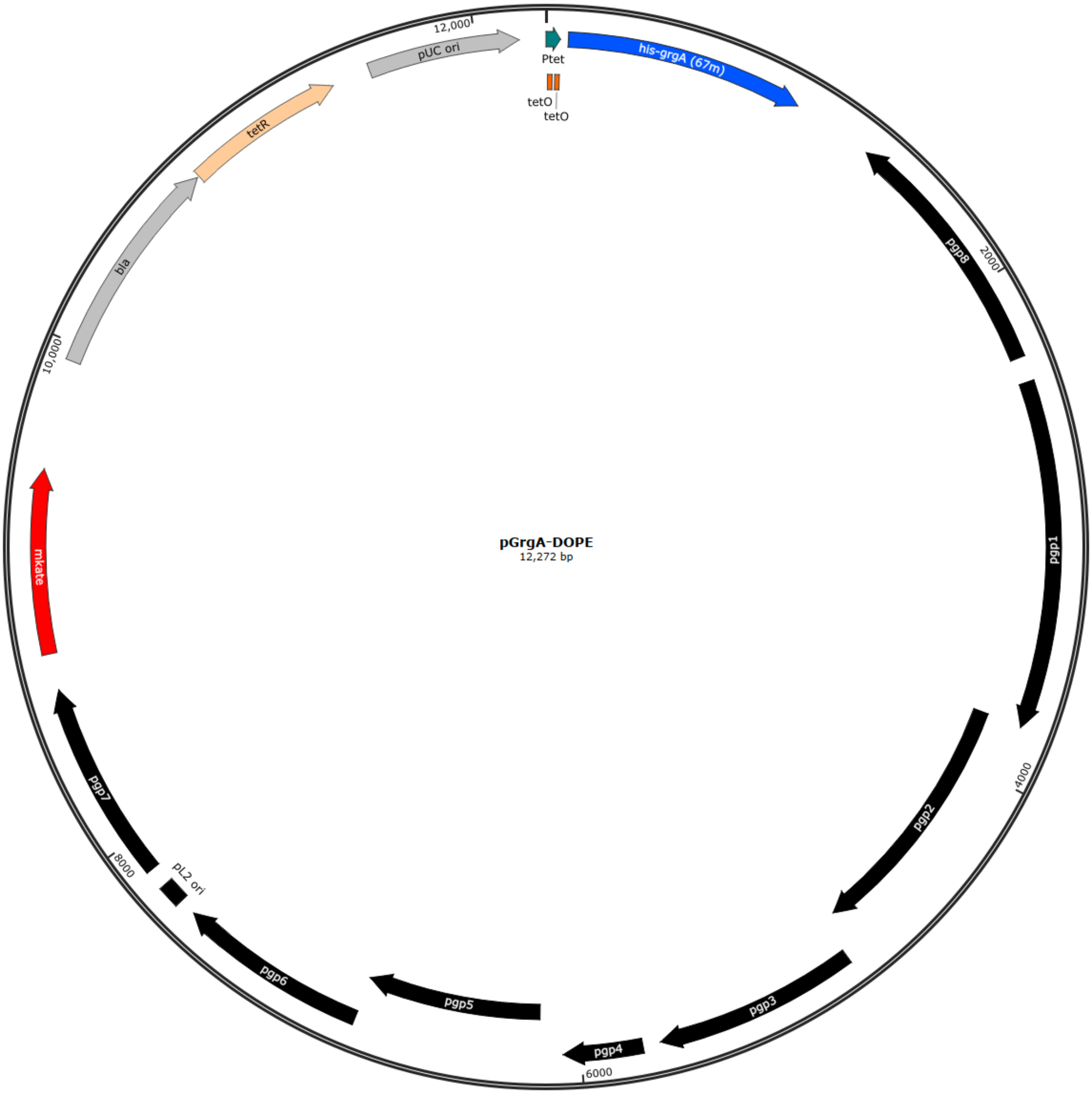

sFigure 1. Map of the shuttle plasmid pGrgA-DOPE. his-grgA (67m) refers to the gene encoding N-terminal His-tagged GrgA that is resistant to Targetron insertion between nucleotides 67 and 68. See Figure 1C for synonymous mutations engineered around this site that rendered the resistance.
